# COVID-19 Resulting in Global Stress Cardiomyopathy in a Young Female

**DOI:** 10.7759/cureus.26779

**Published:** 2022-07-12

**Authors:** Supraja Achuthanandan, Nikhil L Cordeiro, Amit Dhaliwal, Daniel J Masri, Adnan Sadiq, Gerald Hollander

**Affiliations:** 1 Internal Medicine, Maimonides Medical Center, Brooklyn, USA; 2 Cardiology, Maimonides Medical Center, Brooklyn, USA; 3 Radiology, Maimonides Medical Center, Brooklyn, USA

**Keywords:** takotsubo cardiomyopathy, stress induced cardiomyopathy, coronavirus disease 2019 (covid-19), covid 19, inverted takotsubo, takotsubo cardiomyopathy (ttc), transthoracic echocardiogram

## Abstract

Takotsubo cardiomyopathy, also called stress cardiomyopathy, is a form of reversible cardiomyopathy that occurs during periods of emotional or physical stress. There are many variants of takotsubo. They are classified depending on the region of hypokinesis: the most common four variants include the apical/typical variant (left ventricular apical hypokinesis), the midventricular type (midventricular hypokinesis), the basal type (basal hypokinesis), and the focal type (isolated segmental dysfunction of the left ventricle). Rarely takotsubo presents as a global variant where there is global left ventricular hypokinesis. Takotsubo cardiomyopathy has had an increasing incidence since the COVID-19 pandemic. We report a case of a 29-year-old woman with no prior cardiac history who presented with a seizure and was found to have COVID-19. The patient's echocardiogram showed global cardiomyopathy, a rare type of takotsubo cardiomyopathy.

## Introduction

Takotsubo cardiomyopathy (TCM), also called stress cardiomyopathy, is a form of reversible cardiomyopathy that occurs during periods of emotional/physical stress. TCM accounts for 1% to 2% of patients presenting with acute coronary syndrome (ACS) [1,2]. In the apical variant of TCM, the heart takes the appearance of an octopus fishing pot, hence the name takotsubo in Japanese [3]. TCM has become increasingly common among the general population and also patients with COVID-19 [4]. Pathogenesis of TCM is linked to catecholamine surge leading to microvascular dysfunction and coronary spasms, causing myocardial stunning [5,6]. Similarly, the cardiovascular dysfunction seen in coronavirus disease (COVID-19) patients is hypothesized to be related to myocardial toxicity and microvascular dysfunction from elevated cytokines and physical/psychological stressors linked to COVID-19. We report the case of a 29-year-old woman with no prior cardiac history who presented after a seizure and was found to have COVID-19. The patient had no significant coronary artery disease or myocarditis; echocardiography demonstrated global left ventricular (LV) hypokinesis that resolved after weeks of acute illness.

## Case presentation

A 29-year-old woman with a history of bipolar disorder presented with seizure-like activity. The patient was found on the street having generalized limb jerking of both upper and lower extremities. Emergency medical services were called and gave intravenous midazolam, which aborted the seizure-like activity after 20 minutes. Per the patient's family, family history was significant for heart failure, morbid obesity, and uncontrolled diabetes in the patient's mother leading to her death in her 40s. Upon arrival in the emergency department, her vital signs revealed a temperature of 99.8F, blood pressure of 82/32 mmHg, heart rate of 91 beats per minute, and oxygen saturation of 97% on room air. She was minimally responsive and only withdrawing to painful stimuli in all four extremities. Pupils constricted bilaterally and reacted to light. Reflexes were symmetric and 2+ throughout. Auscultation of the heart was unremarkable, and auscultation of the lungs revealed decreased breath sounds at the bases bilaterally. There were no distended neck veins or lower extremity edema. The patient became less responsive and was later intubated for airway protection.

**Figure 1:**
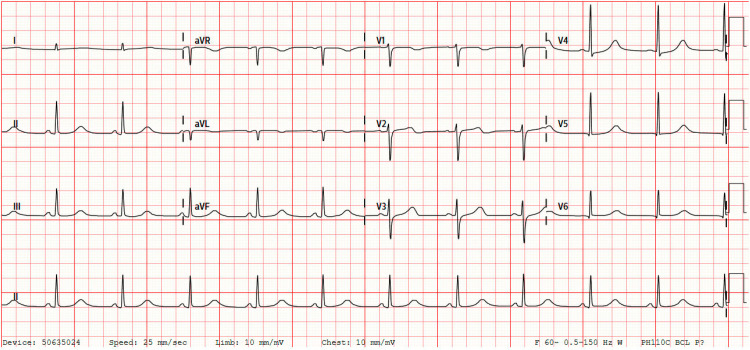
Electrocardiogram at the time of admission showing a normal sinus rhythm with prolonged QTc at 545 ms

**Figure 2:**
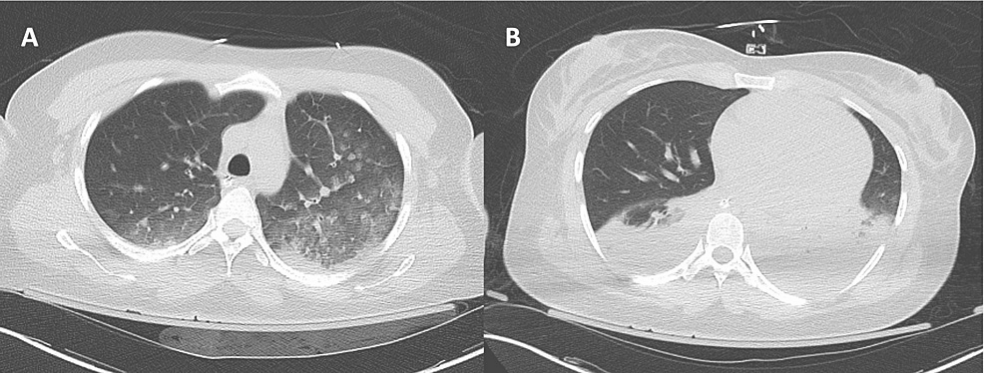
Computed tomography of the chest without contrast A, Ground-glass opacities in the bilateral upper lobes (left greater than right) compatible with pneumonia, including COVID pneumonia. B, Atelectasis/consolidation of the lower lobes, which is also suspicious for pneumonia, including aspiration pneumonia.

Initial blood work showed a white blood cell count of 12.9 K/UL, blood glucose of 170 mg/dL, potassium of 4.8 mmol/L, bicarbonate of 13 mmol/L, blood urea nitrogen of 9.52 mmol/L, creatinine of 1.6 mmol/L, and troponin of 0.47 ng/mL. Venous blood gas revealed a pH of 6.99, partial pressure of carbon dioxide (PCO_2_) of 58.9, and lactic acid of 13.5 mmol/L. The patient was also noted to be COVID positive. The electrocardiogram (EKG) at the time of admission revealed a normal sinus rhythm with a prolonged corrected QT interval (QTc) at 545 ms (Figure 1). Electroencephalography ruled out nonconvulsive status epilepticus. Computed tomography (CT) of the head revealed no acute intracranial pathology but did show encephalomalacia in the right frontal lobe, likely from prior traumatic injury. CT of the chest without contrast revealed ground-glass attenuation and interlobular thickening suggestive of infection or pulmonary edema in the bilateral upper lobes (Figure 2A). The bilateral lower lobes showed consolidations suggestive of aspiration pneumonia (Figure 2B).

**Figure 3:**
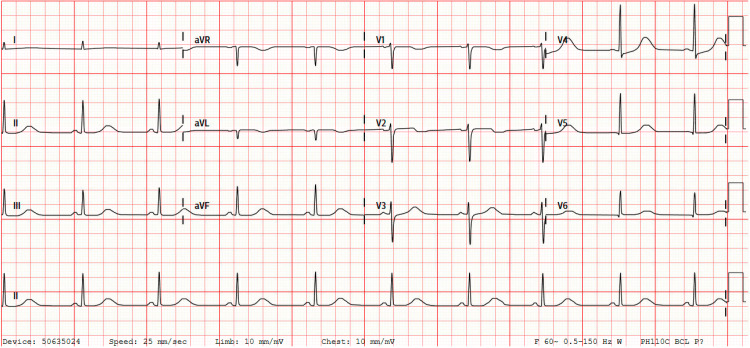
Electrocardiogram during hospitalization which shows prolonged QTc with sinus bradycardia

**Figure 4:**
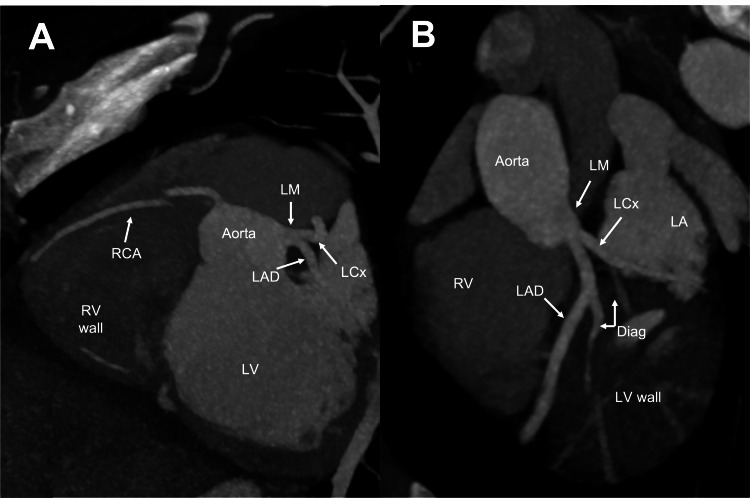
Coronary computed tomography angiography during hospitalization A short left main (LM) artery and right coronary artery (RCA), cut-off due to motion artifact in A, are seen branching off the aorta. The left main (LM) artery bifurcates into the left circumflex artery (LCx) and the left anterior descending artery (LAD). The diagonal branches (Diag) are seen coming off the left circumflex artery (LCx) and the left anterior descending artery (LAD) in B. The coronaries are normal with a zero calcium score. LA: left atrium, RV: right ventricle, LV: left ventricle.

**Figure 5:**
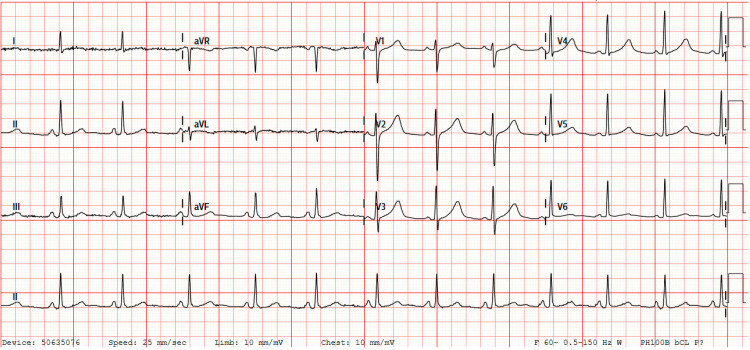
Electrocardiogram two months after hospitalization which shows normal sinus rhythm with the resolution of QTc prolongation

She was admitted to the intensive care unit for the management of status epilepticus and septic shock from COVID-19 pneumonia. Continuous video electroencephalographic monitoring was done, which revealed no electrographic seizures or interictal epileptiform discharges, and no further seizure-like activity was noted during hospitalization. Serial troponins were monitored, peaking at 12.85 ng/mL on day two of hospitalization. She was treated with heparin infusion for possible non-ST elevation myocardial infarction. EKGs continued to show prolonged QTc now with sinus bradycardia (Figure 3). An echocardiogram revealed a left ventricular ejection fraction (LVEF) of 26%-30%, variably hypokinetic to akinetic LV wall motion, normal right ventricular size and systolic function, and mild thickening of anterior and posterior mitral valve leaflets with mild mitral valve regurgitation (Video 1). By day four of hospitalization, the patient was titrated off the vasopressors and extubated to a low-flow nasal cannula. Coronary CT angiography was obtained, revealing normal coronaries with a coronary calcium score of 0 (Figures 4A, 4B). A repeat echocardiogram on day seven of hospitalization had similar findings to the initial echocardiogram from admission. The patient left against medical advice on day eight of hospitalization; she was discharged on losartan and carvedilol and was given a wearable defibrillator and follow-up appointments with neurology and cardiology. A repeat echocardiogram two months posthospitalization revealed normal LV size and wall thickness, with an LVEF of 51%-55% and no wall motion abnormalities and also noted was a normal right ventricular size and function with normal pulmonary artery systolic pressure (Video 2). Cardiac magnetic resonance imaging revealed mild global decreased ventricular wall motion without evidence of myocardial fibrosis, infiltrative process, or inflammation (Video 3). EKG at follow-up showed normal sinus rhythm without QT prolongation (Figure 5).

**Video 1:** Initial transthoracic echocardiogram showing global hypokinesis with LVEF of 26%-30% TTC: takotsubo cardiomyopathy, LVEF: left ventricular ejection fraction.

**Video 2:** Follow-up transthoracic echocardiogram showing a normal EF with resolution of global dysfunction TTC: takotsubo cardiomyopathy, EF: ejection fraction.

**Video 3:** Cardiac MRI showing mild global decreased ventricular wall motion without evidence of myocardial fibrosis, infiltrative process, or inflammation

## Discussion

Takotsubo cardiomyopathy is a syndrome where the heart takes on the appearance of an octopus fishing pot, which is called takotsubo in Japanese. It is known to be associated with emotional/physical stress and thus is also called broken heart syndrome. TCM occurs in approximately 1% to 2% of patients presenting with troponin-positive suspected ACS [1,2]. The various cardiovascular manifestations seen with COVID-19 include venous thromboembolism, arrhythmias, ACS, and myocarditis. TCM can also be seen in patients with severe acute respiratory syndrome coronavirus 2 (SARS-CoV-2) and has been increasingly common since the COVID-19 pandemic [4]. Pathogenesis of TCM is not well understood; however, it is theorized to be due to excess catecholamines leading to microvascular dysfunction and coronary artery spasm/dysfunction resulting in myocardial stunning, appearing similar to segmental wall motion abnormalities in a myocardial infarction but without significant coronary artery disease [5,6].

TCM has multiple variants; the apical variant is the most common and is found in 81.7% of patients in the International Takotsubo Registry study [7]. The less common variants include midventricular type, basal type, and focal type (found in 14.6%, 2.2%, and 1.5% of patients, respectively, in the International Takotsubo Registry study). The apical type has apical ballooning of the LV during systole, with depressed functions of the midsegments and apical segments, and hyperkinesis of the basal segments. The midventricular type is the second common type of TCM; there is midventricular hypokinesis with relative sparing of the apex. The basal type is opposite to the apical type, where there is LV basal hypokinesis with midventricular and apical sparing; hence it is also called reverse/inverted takotsubo. The focal variant is characterized by isolated segmental dysfunction of the LV, most commonly the anterolateral segment. Lastly, global type, a form of TCM involving global hypokinesis, is rarely found; it has been reported in a limited case series in patients with acute medical illness without sepsis/myocarditis [8].

We present a young woman with COVID-19 and non-ST-elevation myocardial infarction, who was found to have transient global LV hypokinesis on echocardiogram. Coronary artery disease (CAD) was not suspected in this young female; CT angiography of the coronary was obtained, and it ruled out significant obstructive CAD. Additionally, she had no evidence of myocarditis on cardiac MRI. The patient was diagnosed with TCM based on these diagnostic criteria proposed by the Mayo Clinic [9,10]. The patient had global hypokinesis instead of regional wall motion abnormalities that resolved after the acute stressor. It is possible that the catecholamine surge from a severe acute stressor would cause not only regional wall motion abnormalities but also global ventricular hypokinesis. Studies with larger power need to be conducted to assess the causation of COVID-19 and global stress cardiomyopathy, as it is possible that this patient's cardiomyopathy was a result of her seizure and severe acidosis instead of COVID-19 directly.

## Conclusions

SARS-CoV-2 can cause many cardiovascular pathologies, which include arrhythmias, acute coronary syndrome, myocarditis, and venous thromboembolism. Apical, basal, and midventricular variants of takotsubo cardiomyopathy are becoming more common among patients with COVID-19. Patients with COVID-19 can also have global cardiomyopathy that is not secondary to myocarditis and is reversible over time following the removal of the stressor.
